# Frequency of Key Type 1 Diabetes Self‐Care Behaviours Are Associated With Psychological Flexibility, Distress and Glycaemic Outcome

**DOI:** 10.1002/edm2.70336

**Published:** 2026-09-19

**Authors:** Jennifer A. Nicholas, Bu B. Yeap, Donna Cross, Natasha Bear, Melanie S. Burkhardt

**Affiliations:** ^1^ Medical School The University of Western Australia Perth Western Australia Australia; ^2^ Department of Endocrinology and Diabetes Fiona Stanley Fremantle Hospitals Group Perth Western Australia Australia; ^3^ Department of Endocrinology and Diabetes Royal Perth Bentley Hospital Group Perth Western Australia Australia; ^4^ School of Global and Public Health The University of Western Australia Perth Western Australia Australia; ^5^ The Kids Research Institute Australia Perth Western Australia Australia; ^6^ Institute for Health Research, Notre Dame University Perth Western Australia Australia; ^7^ Department of Clinical Psychology and Clinical Neuropsychology Fiona Stanley Fremantle Hospitals Group Perth Western Australia Australia; ^8^ Department of Clinical Psychology and Clinical Neuropsychology Royal Perth Bentley Hospital Group Perth Western Australia Australia

**Keywords:** diabetes distress, HbA1C, psychological flexibility, self‐care behaviours, Type 1 diabetes

## Abstract

**Introduction:**

Living with Type 1 diabetes (T1D) requires multiple daily self‐care behaviours. Identifying important self‐care behaviours associated with health and wellbeing has the potential to support a goal‐focussed and person‐centred consultation. We describe diabetes self‐care behaviours and their associations with clinical outcomes including glycated haemoglobin (HbA1C) and diabetes distress, and characterise key behavioural measures associated with poorer outcomes that could be targeted via behavioural intervention.

**Methods:**

Cross‐sectional study involving 105 adults with T1D attending a tertiary diabetes outpatient clinic. Self‐report questionnaires administered during routine appointments assessed diabetes self‐care behaviours, measures of distress and psychological flexibility. Associations between behaviours, HbA1C and distress were examined. Cluster analysis identified subgroups of participants with similar profiles, providing insights into frequency of self‐care behaviours, HbA1C and distress.

**Results:**

Half the participants reported lower frequencies of some key behaviours than generally considered necessary to manage day‐to‐day glucose variations. HbA1C less than 64 mmol/mol (< 8%) was associated with higher frequency of glucose checks, frequency of insulin administration, less prandial insulin omission, and days having hypoglycaemia treatment food available. Clinically significant distress was associated with less frequent insulin administration and less frequent glucose checks before administering insulin. Higher perceived attainment of diabetes self‐care goals and psychological flexibility were associated with lower HbA1C and distress.

**Conclusions:**

A point of care self‐reported questionnaire can identify the frequency of clinically meaningful self‐care behaviours, particularly those most strongly associated with glycaemia and/or distress. This could inform targeted strategies to enhance health outcomes. A prospective study is necessary to test whether routine collection of behavioural information would enhance personalised approaches to diabetes care.

## Graphical abstract

Higher frequency of key diabetes self‐care behaviours, including glucose checks, having insulin with meals, and hypo treatment food available, was associated with lower HbA1c and less diabetes distress. A point‐of‐care questionnaire can identify clinically meaningful patterns in diabetes self‐care to guide personalised consultations with adults living with Type 1 diabetes.

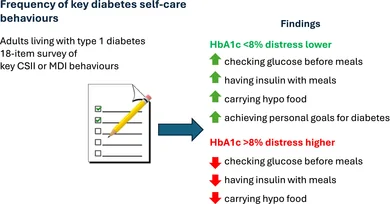

## 1 Introduction

To sustain health and wellbeing, diabetes self‐care behaviours related to the personalised insulin and glucose monitoring regimen underpin the management of Type 1 diabetes (T1D) [1]. Various factors make it complex to regulate constantly changing blood glucose levels, necessary to reduce the risk of health complications related to living with T1D.

Diabetes self‐care behaviours have been broadly categorised into seven key areas: healthy coping; healthy eating; being active; taking medication; monitoring; reducing risks; and problem solving [2]. For people living with T1D, important self‐care behaviours relating to glycaemic management include administering an insulin regimen, through multiple daily injections (MDI) or continuous subcutaneous insulin infusion (CSII), amid rapidly changing diabetes technology.

Patterns of less‐than‐optimal glucose measures are commonly experienced by people with T1D [3, 4]. For the person with T1D and the clinician, individual patterns in diabetes self‐care behaviours can provide an important source of data to identify what is working well and where problems may be arising. Self‐care behaviours can be directly targeted via intervention and therefore it is important for the person with diabetes and clinician to understand how the frequency of key self‐care behaviours may be related to the insulin regimen, glycaemic measures and/or diabetes‐related distress. Understanding these associations could help the person with T1D identify specific self‐care behaviours as targets for change, with the potential to improve glycaemic outcomes and diabetes distress.

Consultations can be time constrained, complex and include an array of glycaemic metrics to consider. Multiple factors must be considered in the context of individual diabetes self‐care behaviours and emotional factors that could inform personalised interventions [5]. Diabetes distress describes the negative emotional impact of living with diabetes [6]. Distress is not uncommon; its source can vary [7] and may be associated with diabetes self‐care, glycaemia [8, 9, 10, 11] and empowerment [12]. Where diabetes distress has been a key focus, interventions have been effective in reducing distress [13, 14]. Sources of distress, examined alongside individual diabetes self‐care behaviours, require further understanding. Currently available tools, however, lack the specificity to measure the frequency of individual diabetes self‐care behaviours unique to adults living with T1D, further supported by evidence that subjective judgements about personal glucose levels are stronger predictors of distress than objective CGM data [15].

Recently published behavioural approaches targeting diabetes distress and HbA1c included strategies and principles to facilitate individuals taking valued actions (i.e., psychological flexibility) over dominance of psychological internal reactions in the moment (i.e., experiential avoidance) and observed beneficial change in self‐care behaviour [16] and distress [16, 17]. Psychological flexibility/inflexibility has also been linked to HbA1c and diabetes distress [16, 18, 19], yet how this relates to diabetes self‐care behaviours requires further investigation. This is important given the role of both as potential mediators of change.

The aim of this research was to explore and characterise associations of behavioural, psychological and knowledge‐related factors in adults with T1D. The self‐reported frequency of key, well‐defined T1D self‐care behaviours, specific to the insulin regimen, and associations with HbA1c and distress are highlighted. Clusters of items associated with greater risk are identified as targets for future behavioural interventions.

## 2 Methods

### 2.1 Study Design and Participants

This cross‐sectional study characterised a range of diabetes self‐care behaviours and their associations with point of care HbA1C and a measure of diabetes distress. The Human Research Ethics Committees of the South Metropolitan Health Service and the University of Western Australia approved the study (RGS501). Over 8 months, a researcher attended multidisciplinary diabetes outpatient clinics at a single tertiary hospital. Participants were aged 18–40 years with a diagnosis of T1D for at least 6 months, independently self‐managing diabetes, did not require an interpreter and were attending a scheduled appointment. All participants provided informed consent, then completed a questionnaire (taking around 15–20 min to complete) comprising a series of self‐report measures, on a single occasion at the time of the scheduled diabetes outpatient clinic appointment.

Point of care HbA1C is routinely collected during a scheduled outpatient clinic at this hospital and was obtained from the medical record at the time the questionnaire was completed. The sociodemographic data collected included formal education, income and private health insurance status. Clinical information including acute complications of diabetes and the patient's attendance at education sessions relating to carbohydrate counting skills were also collected and has previously been reported [18]. This study was completed before a national subsidy (National Diabetes Services Scheme) became available supporting access to continuous glucose monitoring (CGM).

### 2.2 Study Measures

A review of self‐reported diabetes self‐care behaviours was conducted using the Behavioural Diabetes Care Record (BDCR; Data S1) [16]. Items in the BDCR represent what is generally considered necessary to self‐manage the day‐to‐day glucose variations of T1D and routinely taught as part of structured programs or within diabetes consultations. The BDCR takes under 5 min to complete and consists of 18 items; 16 single‐item self‐report measures of common, clinically relevant diabetes self‐care behaviours (fundamental to the T1D medical regimen) occurring in the preceding 14 days and two questions prompting the individual to rate (on a scale of 1–10), the extent to which their personal goals for diabetes self‐care are currently achieved, and willingness to make changes to improve diabetes self‐care. Two versions have been adapted based on insulin regimen, multiple daily insulin injections (MDI) or continuous subcutaneous insulin infusion (CSII). Fourteen of the questions are identical for both versions. The self‐care behaviours measured reflect core information and skills incorporated into diabetes self‐management education programs or individualised consultations, including glucose monitoring, insulin administration, use of personalised insulin to carbohydrate and insulin sensitivity ratios and managing hypoglycaemia. Most of the single item questions describe the self‐care behaviour in observable and measurable terms and require the individual to provide a number to indicate the frequency of the specified behaviour occurring over the preceding 14 days. For example, questions across both versions include ‘On average, how many times per day did you test your blood glucose levels over the past 14 days?*’* ‘On how many of the last 14 days did you eat a main meal without administering insulin?*’ ‘*On how many of the last 14 days did you have your hypo food with you for most of the day?*’*. Questions specific to CSII are included to explore factors that could contribute to glucose variability when actions are delayed, for example ‘On how many of the last 14 days did you re‐site your cannula?’ Similarly, questions specific to MDI explore frequency of times insulin dose was missed and the influence on glucose variability.

These single‐item measures of diabetes self‐care behaviours encompassing the BDCR can be repeated to assess changes in behavioural frequencies over time and in response to behavioural intervention. The measures have been shown to be responsive to behavioural intervention targeting self‐care behaviours [with medium (*p* = 0.008; $\eta_{p}^{2}$ = 0.135) to large (*p* < 0.001; $\eta_{p}^{2}$ = 0.696) effect sizes], and improvements in individual self‐care behaviours also appear to correspond with improvements in HbA1C and distress [16].

The Diabetes Distress Scale (DDS17) is a 17‐item self‐report measure with four sub‐scales, which aggregates to a total score, measuring aspects of diabetes self‐care that may lead to distress (*α* = 0.92) [20]. The sub‐scales relate to emotional burden, physician‐related distress, regimen‐related distress and interpersonal distress [20]. A mean score of three or above can identify distress that could be impacting diabetes self‐care behaviours and is considered clinically important [21] and was the cut point for this analysis.

The Acceptance and Action Questionnaire (AAQ‐II) measures psychological inflexibility and experiential avoidance and has good reliability and validity across different samples and behaviours (mean *α* = 0.84) [22]. Greater psychological inflexibility has been associated with greater depressive, anxiety, and stress‐related symptoms, and the AAQ‐II offers a general measure to examine psychological and behavioural processes, that holds true to its theoretical basis [22]. The AAQ‐II was reverse scored in the current analysis to reflect psychological flexibility. The Behavioural Activation for Depression Scale—Short Form (BADS‐SF) measures activation toward goal‐directed behaviours and reported acceptable internal consistency (*α* = 0.81) [23].

### 2.3 Data Analysis

Primary outcomes reported in this paper include a range of self‐reported diabetes self‐care behaviours (BDCR), a point of care glycaemic measure (HbA1C), distress (DDS17), a measure of psychological flexibility (AAQ‐II) and activation (BADS‐SF). Categorical data are reported as frequencies and percentages, and continuous variables presented as means and standard deviations, unless skewed whereupon medians and interquartile ranges are reported. HbA1C was dichotomised into less than 64 mmol/mol (*≤* 8.0%) and 64 mmol/mol (8.0%) or more, and was determined a priori, in consideration of the proportional risk of diabetes related complications and HbA1C and as a clinically meaningful threshold; distress was dichotomised into < 3, and ≥ 3 representing higher distress and association with glycaemia has previously been reported [21]. Associations between the outcomes of HbA1C and distress, and diabetes self‐care behaviours were analysed using the Mann Whitney *U* test. Latent class cluster analysis was then used to detect subgroups with similar profiles within this population. Items common across the two BDCR versions were considered for cluster analysis; selection was determined through review of the literature, consultation with two consumers with lived experience of T1D (with assistance from the University of Western Australia, Consumer and Community Involvement Program), diabetes specialist clinicians from medical, nursing and nutrition disciplines, and considering clinical and statistical significance. Bayesian Information Criterion, class size (no < 5%), entropy and interpretability were used to determine the number of classes and model fit. Statistical significance was set at *p* < 0.05. All statistical analysis was performed using Stata v 18.1 (StataCorp, College Station, Texas).

## 3 Results

### 3.1 Participants

Characteristics of the participants are presented in Table 1. In total, 115 people were identified as eligible and approached, 108 agreed and seven declined to participate. Of the 105 participants who completed the questionnaire, most (64%) reported they had received formal carbohydrate counting instruction or had seen a diabetes specialist Dietitian to learn about carbohydrate counting, and 29% reported using a form of CGM (*n* = 30).

**TABLE 1:** Demographic and clinical characteristics of the sample group (*n* = 105).

Male: *n* (%)	56 (53%)
Age (years)	
mean (SD)	27 (7.1)
Diabetes duration (yrs.) mean (SD)	12.6 (8.5)
min – max (years)	0.5–35
Glycated haemoglobin %	
mean (SD)	8.7 (2.0)
range: min—max	5.6–14.0
Glycated haemoglobin mmol/L	
mean	72
range: min—max	38–130
Diabetes distress	
Median (IQR)	2 (1,2)
Range: min—max	1–4
Multiple daily insulin injections (MDI) *n* (%)	69 (66)
Continuous subcutaneous insulin infusion (CSII) *n* (%)	36 (34)

### 3.2 Diabetes Self‐Care Behaviours and Associations With HbA1C and Diabetes Distress

Participants self‐reported diabetes self‐care behaviours for the previous 14 days and key items and associations with HbA1C and distress are included in Table 2. Frequency of glucose checks (median 4 per day, IQR 2–6) was associated with HbA1C less than 64 mmol/mol (< 8%), compared with three checks per day (IQR 2–4; *p* = 0.044). Days on which prandial insulin was missed were also associated with HbA1c, with a median of no days missed (IQR 0–2) compared with 2 days in the preceding 14 days (IQR 0–6; *p = 0*.001).

**TABLE 2:** Behavioural Diabetes Care Record (BDCR) items and associations with HbA1C and diabetes distress.

HbA1C					DDS		
BDCR items self‐reported for the preceding 14 days	*n*	HbA1C < 64 mmol/mol [< 8%; Median (IQR)]	HbA1C ≥ 64 mmol/mol [≥ 8%; Median (IQR)]	Rank sum test *p* value	DDS < 3 Median (IQR)	DDS 3+Median (IQR)	Rank sum test *p* value
Days BG checked min. 1×/day^a^	103	14 (14, 14)	14 (12, 14)	*p* = 0.168	14 (14, 14)	14 (5, 14)	*p* = 0.117
Frequency BG checks per day	101	4 (2, 6)	3 (2, 4)	*p* = 0.044	3 (2, 5)	3 (1, 5)	*p* = 0.587
Days CSII suspend/disconnect > 30 min	35	0 (0, 3)	2 (0, 5)	*p* = 0.200	0 (0, 5)	4 (1, 8)	*p* = 0.150
MDI, days insulin admin. min. 1×/day	65	14 (14, 14)	14 (14, 14)	*p* = 0.844	14 (14, 14)	14 (14, 14)	*p* = 0.900
CSII, days reservoir changed	34	4 (2, 4)	4 (4, 5)	*p* = 0.214	4 (3, 4)	4 (4, 4)	*p* = 0.378
MDI, frequency insulin admin. per day	64	4 (4, 5)	4 (3, 4)	*p* = 0.038	4 (3, 5)	3 (3, 4)	*p* = 0.026
CSII, days infusion line changed	34	4 (3, 4)	4 (3, 5)	*p* = 0.330	4 (3, 4)	4 (4, 6)	*p* = 0.240
MDI, days insulin missed min. 1×/day	64	0 (0, 2)	1 (0, 4)	*p* = 0.137	0 (0, 2)	3 (1, 14)	*p* = 0.005
CSII, site/cannula change	34	4 (3, 4)	4 (3, 5)	*p* = 0.595	4 (3, 4)	4 (4, 6)	*p* = 0.389
MDI, days admin. long‐acting insulin	65	14 (14, 14)	14 (14, 14)	*p* = 0.787	14 (14, 14)	14 (14, 14)	*p* = 0.615
Days prandial insulin missed (main meal)^a^	101	0 (0, 2)	2 (0, 6)	*p* = 0.001	1 (0, 2)	3 (0, 7)	*p* = 0.015
Days prandial insulin missed (snack)	100	2 (0, 3)	3 (0, 7)	*p* = 0.231	2 (0, 3)	5 (3, 10)	*p* < 0.001
Count carbohydrate (% times)	96	70 (19, 98)	50 (0, 80)	*p* = 0.151	60 (10, 90)	50 (0, 80)	*p* = 0.352
BG check prior to meals (% times)^a^	100	80 (50, 100)	50 (20, 90)	*p* = 0.059	78 (50, 100)	50 (10, 80)	*p* = 0.032
Total daily insulin dose	88	45 (30, 60)	50 (38, 61)	*p* = 0.457	50 (31, 62)	45 (36, 61)	*p* = 0.816
Estimate % use of ISF	90	20 (0, 60)	30 (10, 80)	*p* = 0.405	28 (1, 80)	20 (0, 40)	*p* = 0.302
Estimate % use of ICR for main meals	89	80 (15, 100)	50 (0, 100)	*p* = 0.255	80 (0, 100)	35 (0, 90)	*p* = 0.248
Estimate % use of ICR for snack	87	65 (0, 90)	30 (0, 90)	*p* = 0.558	60 (0, 100)	15 (0, 70)	*p* = 0.134
Days carrying hypo food^a^	103	14 (12, 14)	13 (4, 14)	*p* = 0.044	14 (10, 14)	14 (7, 14)	*p* = 0.996
Hypo's felt or tested	101	4 (2, 6)	2 (0, 4)	*p* = 0.009	2 (1, 4)	1 (0, 4)	*p* = 0.086
Perceived achievement of DSM goals^a^	100	7 (6, 8)	5 (2, 7)	*p* < 0.001	6 (5, 8)	2 (1, 5)	*p* < 0.001
Willing to improve DSM	100	8 (7, 10)	8 (6, 10)	*p* = 0.758	8 (7, 10)	8 (6, 10)	*p* = 0.456
Psychological flexibility (AAQ‐ II)	101	40 (33.5, 47)	36 (28, 44)	*p* = 0.020	41 (33, 46)	30 (24, 35)	*p* < 0.001
Behavioural activation (BADS‐SF)	101	18 (15, 24)	16 (11, 22)	*p* = 0.116	18 (13, 23)	18 (12, 25)	*p* = 0.984

Abbreviations: BG, blood glucose; CSII, continuous subcutaneous insulin pump therapy; MDI, multiple daily insulin injections; ISF, insulin sensitivity factor; ICR, insulin to carbohydrate ratio; HF, hypoglycaemic treatment food; DSM, diabetes self‐management.

^a^ Items identified as clinically important.

For participants prescribed a MDI insulin regimen, more frequently administering insulin, with a median of four times per day (IQR 4–5; *p* = 0.038) was associated with HbA1C less than 64 mmol/mol (< 8%). Participants with an HbA1C less than 64 mmol/mol (< 8%) were more likely to report being equipped to manage hypoglycaemia and felt or checked for hypoglycaemia more frequently with a median of four (IQR 2–6), compared to only two times (IQR 0–4) for those with an HbA1C greater than 64 mmol/mol (≥ 8%, *p* = 0.009). Notably, clinically significant distress was associated with a greater frequency of days where insulin was missed for main meals (*p* = 0.015) and snacks requiring insulin (*p* < 0.001). Diabetes distress rated at a level considered clinically important was also associated with less frequent glucose checks before meals (*p* = 0.032). Perceived achievement of diabetes self‐care goals and psychological flexibility were significantly associated with lower HbA1C and less distress (see Table 2).

### 3.3 Clusters of Diabetes Self‐Care Behaviours and Associations With HbA1C and Diabetes Distress

A cluster analysis (Table 3) identified three subgroups showing patterns of association between key diabetes self‐care behaviours, HbA1C, and distress. A half of participants (Cluster 1, 50%) were more likely to report regularly checking glucose every day, before administering insulin, and fewer days where prandial insulin was missed. They were also least likely to have a HbA1C greater than 64 mmol/mol (≥ 8%) or to report higher distress. At the same time, measures of psychological flexibility and achieving personal goals for T1D were rated higher.

**TABLE 3:** Cluster profile of diabetes self‐management behaviours and associations with HbA1C, diabetes distress, psychological flexibility.

Clusters	Marginal Probabilities	Associations of diabetes self‐management, HbA1C, distress (DDS) and psychological flexibility (AAQ‐II, BADS‐SF)	Measure	Probabilities/Mean
Cluster 1 Self‐reported higher frequency of key diabetes self‐care behaviours	49.6%	Days BG checked min. 1×/day (< 14 = no, 14 = Yes)	%	91%
		Days hypoglycaemia treatment available most of day (< 14 = no, 14 = Yes)	%	62%
		HbA1C ≥ 64 mmol/mol (≥ 8%; < 8 = no, 8 + = Yes)	%	30%
		DDS 3 + (< 3 = no, 3 + = Yes)	%	4%
		AAQI‐II	Mean	41.1
		BADS‐SF	Mean	19.1
		Days prandial insulin missed (main meal)	Mean	1.9
		BG check prior to meals (% times over 14 days)	Mean	76.5
		Perceived achievement of DSM goals	Mean	7.3
Cluster 2 Self‐reported a moderate frequency of key self‐care behaviours	32.6%	Days BG checked min. 1×/day (< 14 = no, 14 = Yes)	%	76%
		Days hypoglycaemia treatment available most of day (< 14 = no, 14 = Yes)	%	53%
		HbA1C ≥ 64 mmol/mol (≥ 8%; < 8 = no, 8 + = Yes)	%	72%
		DDS 3+ (< 3 = no, 3 + = Yes)	%	29%
		AAQ‐II	Mean	34.8
		BADS‐SF	Mean	16.3
		Days prandial insulin missed (main meal)	Mean	2.9
		BG check prior to meals (% times over 14 days)	Mean	55.2
		Perceived achievement of DSM goals	Mean	4.8
Cluster 3 Self‐reported lower frequency of key self‐care behaviours	17.8%	Days BG checked min. 1×/day (< 14 = no, 14 = Yes)	%	36%
		Days hypoglycaemia treatment available most of day (< 14 = no, 14 = Yes)	%	42%
		HbA1C ≥ 64 mmol/mol (≥ 8%; < 8 = no, 8+ = Yes)	%	99%
		DDS 3 + (< 3 = no, ≥ 3 = Yes)	%	72%
		AAQI‐II	Mean	28.3
		BADS‐SF	Mean	15.5
		Days prandial insulin missed (main meal)	Mean	5.8
		BG check prior to meals (% times over 14 days)	Mean	26.4
		Perceived achievement of DSM goals	Mean	1.2

Abbreviations: AAQ‐II, Acceptance and Action Questionnaire‐II; BADS‐SF, Behavioural Activation Scale—Short Form; BG, blood glucose; DDS, Diabetes Distress Scale; DSM, Diabetes self‐management.

Of participants in cluster two (32.6%), three‐quarters reported checking their glucose level at least once per day, had a HbA1C greater than 64 mmol/mol (≥ 8%), and some reported clinically significant levels of distress and less psychological flexibility. There were a moderate number of days in which prandial insulin was missed, and moderate scores were reported for achieving personal goals related to T1D. Participants in Cluster 3 (18%) were most likely to have a HbA1C > 64 mmol/mol (≥ 8%), reported the highest levels of distress, and were more likely to report a lower rating for achieving personal goals related to diabetes and psychological flexibility. They were less likely to check glucose levels every day and have treatment for hypoglycaemia available. They reported more days where they had a main meal without administering insulin and the lowest percentage of days checking glucose before administering prandial insulin.

## 4 Discussion

In this sample of adults with T1D, self‐reported frequencies of key T1D diabetes self‐care behaviours were associated with HbA1C and distress. This study highlights the importance of identifying critical diabetes self‐care behaviours related to the T1D insulin regimen and determining their associations with glycaemia, diabetes distress and psychological flexibility. A cluster analysis provided further insights into these associations with three identified subgroups of participants. The subgroups identified different patterns of engagement in key self‐care behaviours promoted in diabetes self‐management education, [2] and are clinically relevant due to their associations with HbA1C, diabetes distress and psychological flexibility.

Other studies utilising self‐report questionnaires that measure self‐care as a construct consisting of a range of activities that relate more globally to the diabetes management regimen [24, 25, 26] and those using diabetes technology to provide proxy measures of self‐care behaviour [27, 28] have observed associations with glycaemic outcomes. Whilst these observations align with our findings, the benefit of the single‐item measures provided specificity and allowed investigation of how frequencies of key self‐care behaviours relate to HbA1C, distress, flexibility and the personal goals of individuals with T1D. For example, in the current research, 50% of participants (clusters two and three) reported a lower frequency of behaviours important to effectively manage day‐to‐day glucose variations. Furthermore, a level of distress considered clinically significant was also associated with lower reported frequency of these self‐care behaviours. When applied in diabetes consultations, single‐item T1D self‐care behaviours could be considered alongside other important metrics (e.g., HbA1C, CGM). By triangulating self‐reported data with complementary clinical data, this can mitigate limitations associated with self‐report measures. Together, this integrated information can guide the development of a more personalised and targeted approach, such as tailored goals, options for diabetes technology, or timely referral to other members of the multidisciplinary team (e.g., Dietetic input, Clinical Psychology). The BDCR has previously been utilised for this purpose in the context of a personalised group‐based behavioural intervention [16].

In the current study, how participants rated their achievement of personal diabetes self‐care goals (i.e., higher, moderate or lower) was significantly associated with both HbA1C and distress. Clinically significant distress scores and HbA1C greater than 64 mmol/mol (≥ 8%) were also associated with a lower reported rating for achieving personal goals for diabetes self‐care, and lower psychological flexibility. For individuals who perceive they are falling short of their personal goals for diabetes and/or experience diabetes distress, it is important to evaluate specific diabetes self‐care behaviours that may be impacting health and wellbeing. This would be in line with behavioural approaches to intervention, where the focus of assessment and intervention are personal behaviours in everyday life contexts. We previously observed an association of higher psychological flexibility and lower glycated haemoglobin (HbA1C) and less diabetes distress [18]. The findings of this study further add to the published literature on psychological flexibility, specifically the association of flexibility with clinically relevant diabetes outcomes for adults with T1D and Type 2 diabetes [19].

The cluster analysis highlighted that clinically significant distress may be present even when HbA1C was not especially elevated. This is not surprising considering measures of diabetes distress and HbA1C are separate entities, and sources and levels of distress can fluctuate over time [7]. Understanding the sources or drivers of diabetes distress, alongside glycaemic metrics [15] and person‐specific individual diabetes self‐care behaviours could support a person‐centred approach. Despite all study participants having access to a specialist multidisciplinary diabetes team, these findings further highlight that people living with T1D are managing a complex condition with many influencing factors.

A systematic review of behavioural interventions targeting diabetes distress indicated generally positive changes related to emotional health [29], yet how this influences diabetes self‐care behaviours and glycaemic patterns requires further clarification. Given individual self‐care behaviours underpin the management of T1D, the American Diabetes Association Standards of Care outline a multifaceted approach to support positive health behaviours and psychological well‐being through person‐centred collaborative care [30]. To achieve this, frameworks and diabetes self‐management programs [1, 2] can facilitate the necessary knowledge, practical skills, and access to diabetes technology most suitable for each person's current needs and circumstances. This is particularly important at significant timepoints, including when diabetes is diagnosed, if treatment targets are not being met, at times of change or transition in life, and/or if complications develop [30]. A person‐centred approach includes considering individual goals, strengths, and vulnerabilities [2, 5, 31]. In addition, everyday routines and situations including social contexts can have an influence on diabetes self‐care and may (positively or negatively) impact clinical outcomes. In this research, lower HbA1C and fewer reports of high distress were associated with more frequent glucose checks, more consistent administration of prandial insulin, and having hypoglycaemia treatment readily available throughout the day (cluster one: Table 3). The variable frequency reported for some of these activities considered clinically important illustrates the difficulties people may experience managing this complex health condition.

### 4.1 Strengths and Limitations

Findings from this study illustrate the value of an easily administered point of care questionnaire that specifically targets diabetes self‐care behaviours to guide a conversation to provide a more person‐centred approach for adults living with T1D.

In addition to evaluating diabetes distress, simple practical measures to document consistency of key individual diabetes self‐care behaviours are clinically relevant and reflects core principles of diabetes self‐management education.

Utilising a self‐report measure, there is the possibility of recall inaccuracies representing a potential limitation of the study. Social desirability bias has the potential to influence reported frequency of self‐care behaviours.

The cross‐sectional study design is a limitation of the study. While the researcher attended various weekly consecutive clinics over 8 months, people who did not attend scheduled appointments or who attended a phone or telehealth appointment may not have had the opportunity to be approached for this study. Associations found with this cohort attending a tertiary centre may not be generalisable to wider populations.

### 4.2 Implications of the Research

- Assessing the frequency of key diabetes self‐care behaviours may help evaluate risk linked to glycaemia and diabetes distress
- Key items in this study associated with higher HbA1C and/or diabetes distress could be explored as a routine measure during consultations with adults with T1D.

## 5 Conclusion

This study identified a potentially at‐risk population, using measures of self‐reported diabetes self‐care behaviours and investigating associations with HbA1C, distress and psychological flexibility. Characterising key aspects of diabetes self‐care specifically related to the insulin regimen offers new insight into associations with HbA1C and distress providing a foundation for more person‐centred, goal orientated consultations.

Notwithstanding the technological advancements with continuous glucose monitoring and hybrid closed loop insulin pump systems, the key diabetes self‐care behaviours identified in this study continue to be recommended as part of standard care, including checking glucose level (or CGM directional trend arrow) before administering insulin, administering or ‘announcing’ a mealtime insulin bolus, and hypoglycaemia treatment being readily available. The findings from this study have the potential to guide more focussed attention and resource allocation to support a more personalised and behavioural approach to improve health and wellbeing for people living with T1D. Incorporating these insights into day‐to‐day T1D consultations may be particularly helpful when glucose levels are not optimal and/or distress is present. A prospective study is warranted to test whether routine behavioural data collection enhances diabetes consultations.

## Author Contributions

**Jennifer A. Nicholas:** conceptualization, investigation, writing – original draft, methodology, formal analysis, project administration, writing – review and editing. **Bu B. Yeap:** conceptualization, writing – review and editing, supervision. **Donna Cross:** writing – review and editing, supervision, conceptualization. **Natasha Bear:** formal analysis, writing – review and editing. **Melanie S. Burkhardt:** conceptualization, writing – review and editing, methodology, supervision.

## Funding

This research was supported by a seeding grant from the Children's Diabetes Centre, Telethon Kid's Institute, Australia.

## Ethics Statement

The Human Research Ethics Committees of the South Metropolitan Health Service and the University of Western Australia approved the study (RGS501).

## Consent

All participants provided informed consent. We have obtained written informed consent from study participants to publish findings from this research. This research was supported by a seeding grant from the Children's Diabetes Centre, Telethon Kid's Institute, Australia. The datasets generated during the current study are available from the corresponding author on reasonable request.

## Conflicts of Interest

The authors have declare no conflicts of interest.

## Supporting information


**Data S1:** Supporting Information.

## Data Availability

The data that support the findings of this study are available from the corresponding author upon reasonable request.
