# Supplementary material for: Frequency of Key Type 1 Diabetes Self‐Care Behaviours Are Associated With Psychological Flexibility, Distress and Glycaemic Outcome
Source: Endocrinol Diabetes Metab. 2026 Sep 19;9(5):e70336. doi: 10.1002/edm2.70336 (PMC13589303; doi:10.1002/edm2.70336)
Supplement: Supplementary file 1 [file EDM2-9-e70336-s001.docx]

# BDCR – CSII

## COMPLETE ONLY IF YOU ARE ON A PUMP*,* OTHERWISE USE THE FORM ON THE NEXT PAGE

The following questions are about your diabetes care routines over the past 14 days. Please read each question carefully, as some questions have similar wording. Please answer all questions with numbers not words. If you are unsure of the exact answer to a question, give your best guess.

| ***Blood glucose testing*** | | | |
| --- | --- | --- | --- |
| 1. | On how many of the last 14 days did you test your blood glucose levels at least once? |  | out of 14 days |
| 2. | On average, how many ***times per day*** did you test your blood glucose levels over the past 14 days? |  | times per day |

| ***Insulin administration*** | | | |
| --- | --- | --- | --- |
| 3. | On how many of the last 14 days did you ***suspend OR disconnect*** your insulin pump for any period of time greater than or equal to 30 minutes? |  | out of 14 days |
| 4. | On how many of the last 14 days did you change the reservoir of your pump? |  | out of 14 days |
| 5. | On how many of the last 14 days did you change your infusion lines? |  | out of 14 days |
| 6. | On how many of the last 14 days did you resite your cannula? |  | out of 14 days |
| 7. | On how many of the last 14 days did you eat a **main meal** without bolusing insulin? |  | out of 14 days |
| 8. | On how many of the last 14 days did you have a **snack** that required insulin, but did not bolus insulin? |  | out of 14 days |
| 9. | When you bolused insulin over the last 14 days, approximately what percentage of times did you **count carbohydrates** first? |  | % |
| 10. | When you bolused insulin over the last 14 days, approximately what percentage of times did you check your **blood glucose levels** first? |  | % |
| 11. | What was your average total daily dose of insulin over the last 14 days? |  | units |
| 12. | Estimate the percentage of times you used your **insulin sensitivity factor**  (correction dose) to correct your blood glucose levels over the last 14 days. | % | |
| 13. | Estimate the percentage of time you used a **carbohydrate-to-insulin ratio** over the last 14 days for your   - ***main meals*** - ***snacks requiring insulin*** |  | %  % |

| ***Hypoglycaemic episodes*** | | | |
| --- | --- | --- | --- |
| 14. | On how many of the last 14 days did you have your hypo food with you for most of the day? |  | out of 14 days |
| 15. | Over the **last 14 days**, how many hypos did you have (include the ones you felt and/or blood glucose level <4mmol/l)? |  | hypos |

| ***Other*** | | | |
| --- | --- | --- | --- |
| 16. | On a scale of 1 to 10 (1 = not achieved and 10 = fully achieved), rate the extent to which your personal goals for diabetes self-care are currently achieved? |  | out of 10 |
| 17. | On scale of 1 to 10 (1 = not at all and 10 = very willing), rate the extent to which you are willing to make changes to improve your diabetes self-care? |  | out of 10 |
| Reference: M.S. Burkhardt, C. Pirri, M.A. Summers, P. Barrie, M. Aghabozorgi, P.G. Fegan, B.B. Yeap. Group-based Behaviour Therapy Improves Self-Care, Glycaemic Control and Distress in Adults with Type 1 Diabetes. Diab Res & Clinical Prac. 208(6) (2024). doi:10.1016/j.diabres.2024.111095 | | | |

## BDCR – MDI

## COMPLETE IF YOU ARE ON MULTIPLE DAILY INJECTIONS

The following questions are about your diabetes care routines over the past 14 days. Please read each question carefully, as some questions have similar wording. Please answer all questions with numbers not words. If you are unsure of the exact answer to a question, give your best guess.

| ***Blood glucose testing*** | | | |
| --- | --- | --- | --- |
| 1. | On how many of the last 14 days did you test your blood glucose levels at least once? |  | out of 14 days |
| 2. | On average, how many ***times per day*** did you test your blood glucose levels over the past 14 days? |  | times per day |

| ***Insulin administration*** | | | |
| --- | --- | --- | --- |
| 3. | On how many of the last 14 days did you administer insulin at least once? |  | out of 14 days |
| 4. | On average, how many ***times per day*** did you administer insulin over the past 14 days? |  | times per day |
| 5. | On how many of the last 14 days did you **miss** an insulin dose at least once? |  | out of 14 days |
| 6. | On how many of the last 14 days, did you administer **long-acting** insulin? |  | out of 14 days |
| 7. | On how many of the last 14 days did you eat a **main meal,** without administering insulin? |  | out of 14 days |
| 8. | On how many of the last 14 days did you have a **snack** that required insulin, but did not administer insulin? |  | out of 14 days |
| 9. | When you administered insulin over the last 14 days, approximately what percentage of times did you **count carbohydrates** first? |  | % |
| 10. | When you administered insulin over the last 14 days, approximately what percentage of times did you check your **blood glucose levels** first? |  | % |
| 11. | What was your average total daily dose of insulin (including your rapid/bolus insulin and long acting/basal insulin added together) over the last 14 days? | units | |
| 12. | Estimate the percentage of times you used your **insulin sensitivity factor**  (correction dose) to correct your blood glucose levels over the last 14 days. | % | |
| 13. | Estimate the percentage of time you used a **carbohydrate-to-insulin ratio** over the last 14 days for your   - ***main meals*** - ***snacks requiring insulin*** |  | %  % |

| ***Hypoglycaemic episodes*** | | | |
| --- | --- | --- | --- |
| 14. | On how many of the last 14 days did you have your hypo food with you for most of the day? |  | out of 14 days |
| 15. | Over the **last 14 days**, how many hypos did you have (include the ones you felt and/or blood glucose level <4mmol/l)? |  | hypos |

| ***Other*** | | | |
| --- | --- | --- | --- |
| 16. | On a scale of 1 to 10 (1 = not achieved and 10 = fully achieved), rate the extent to which your personal goals for diabetes self-care are currently achieved? |  | out of 10 |
| 17. | On scale of 1 to 10 (1 = not at all and 10 = very willing), rate the extent to which you are willing to make changes to improve your diabetes self-care? |  | out of 10 |
| Reference: M.S. Burkhardt, C. Pirri, M.A. Summers, P. Barrie, M. Aghabozorgi, P.G. Fegan, B.B. Yeap. Group-based Behaviour Therapy Improves Self-Care, Glycaemic Control and Distress in Adults with Type 1 Diabetes. Diab Res & Clinical Prac. 208(6) (2024). doi:10.1016/j.diabres.2024.111095 | | | |
